# Supplementary material for: Ex Vivo Antiplatelet and Thrombolytic Activity of Bioactive Fractions from the New-Fangled Stem Buds of Ficus religiosa L. with Simultaneous GC-MS Examination
Source: Molecules. 2023 May 5;28(9):3918. doi: 10.3390/molecules28093918 (PMC10179924; doi:10.3390/molecules28093918)
Supplement: Supplementary file 1 [file molecules-28-03918-s001.zip › molecules-2323527-supplementary.pdf]

Sample Information

Analyzed by	: $Admn.$
Analyzed	: 1/22/2022 10:54:13 PM
Sample Type	: $Organic$
Sample Name	: PI
Method File	: D:\GCMS\Method File\Extract.qgm

Chromatogram E:\Extract\23-1-22\PI.qgd

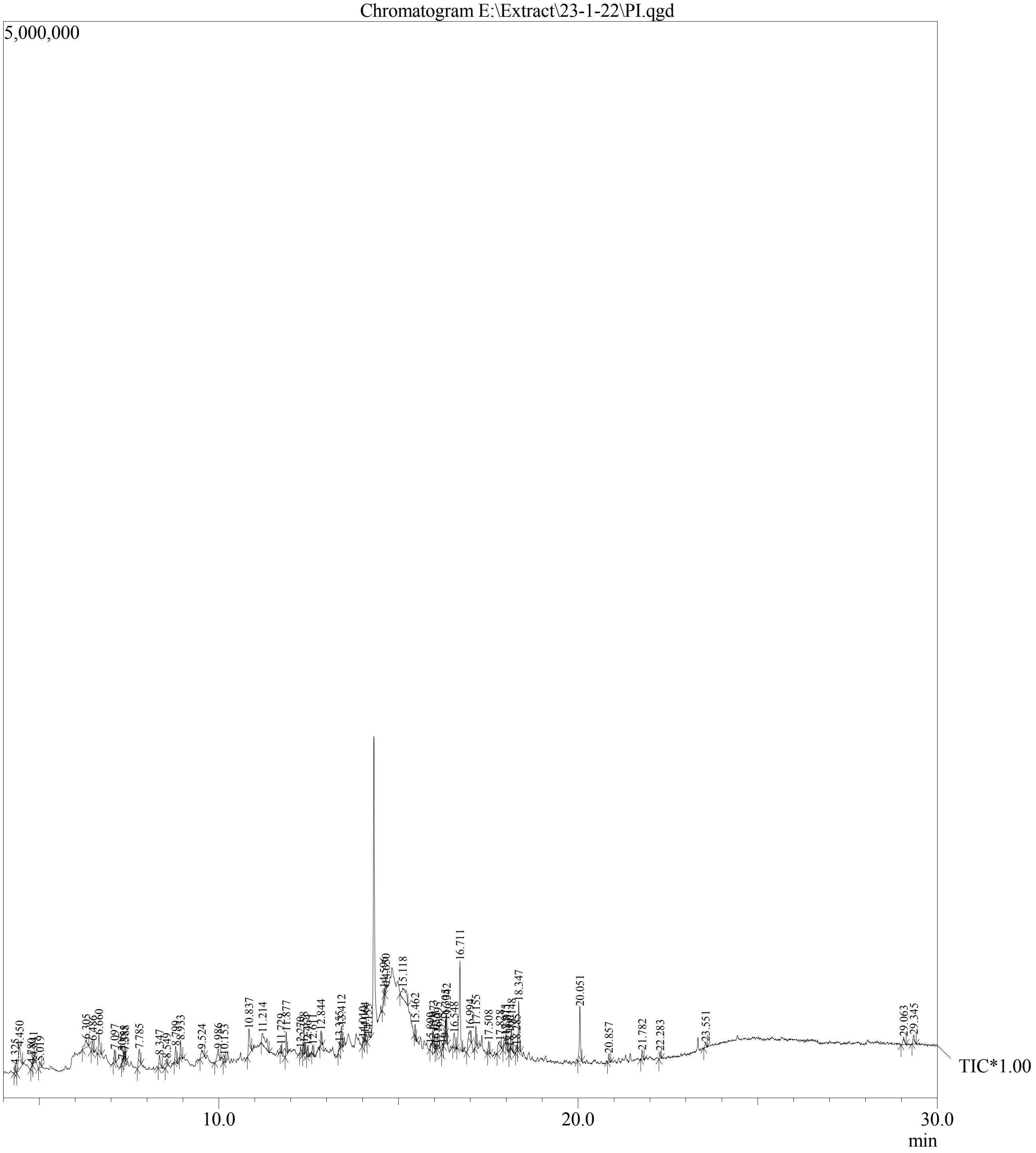

Peak Report TIC

Peak#	R.Time	Area	Area%	Name
1	4.325	52775	0.52	3-Methylcyclopentyl acetate
2	4.450	505536	5.01	2-HYDROXYPROPANOIC ACID
3	4.780	36041	0.36	2-METHYL-5-ISOPROPENYLFURAN
4	4.841	38299	0.38	N-(2-Methylbutylidene)isobutylami
5	5.019	66673	0.66	3-METHYLENEDIHYDRO-2,5-FURANDIONE
6	6.305	178029	1.77	1,2,3,4-Butanetetrol, [S-(R*,R*)]-
7	6.486	113406	1.12	1-BUTANAMINE, 2-METHYL-N-(2-METHYLBUTYLID
8	6.660	242790	2.41	BENZENEACETALDEHYDE
9	7.097	111867	1.11	Ethyl 2-(5-methyl-5-vinyltetrahydrofuran-2-yl)propan-2-yl
10	7.305	24606	0.24	4-HEPTANOL, 4-ETHYL-2,6-DIMETHYL-
11	7.355	42267	0.42	4-HEXEN-1-OL, 5-METHYL-2-(1-METHYLETHENYL)-
12	7.388	33189	0.33	1-(1-METHYLVINYL)-4-METHYL-BENZENE
13	7.785	256235	2.54	Butanedioic acid, monomethyl ester
14	8.347	177878	1.76	4H-Pyran-4-one, 2,3-dihydro-3,5-dihydroxy-6-methyl-
15	8.549	68729	0.68	Dehydromevalonic lactone
16	8.799	179660	1.78	BENZOIC ACID
17	8.933	150264	1.49	3,5-Dimethyl-3-heptene
18	9.524	148895	1.48	2-Hexanol, 2,5-dimethyl-, (S)-
19	9.986	190665	1.89	Propanedioic acid, phenyl-
20	10.153	57208	0.57	2-FURANMETHANOL, 5-ETHENYLTETRAHYDRO-.A
21	10.837	377139	3.74	BENZOIC ACID, 2-HYDROXY-
22	11.214	418919	4.16	2-Acetamido-2-deoxymannosonic acid
23	11.729	47363	0.47	5,8-DECADIEN-2-ONE, 5,9-DIMETHYL-, (E)-
24	11.877	200208	1.99	3-Acetoxy-2(1H)-pyridone
25	12.270	58033	0.58	1-Cyclopentene-1-carboxylic acid, 2-methyl-3-vinyl-
26	12.358	78920	0.78	DL-Norvaline, N-[(phenylmethoxy)carbonyl]-
27	12.464	78312	0.78	2-Propenoic acid, 3-phenyl-
28	12.611	70928	0.70	4-(1,3,3-Trimethyl-bicyclo[4.1.0]hept-2-yl)-but-3-en-2-one
29	12.844	107506	1.07	2,5-Hexanediol, 2,5-dimethyl-
30	13.355	65527	0.65	Phenol, 2,5-bis(1,1-dimethylethyl)-
31	13.412	150183	1.49	7-Oxabicyclo[4.1.0]heptan-3-ol, 6-(3-hydroxy-1-butenyl)-1
32	14.010	43745	0.43	Phenol, 4-ethenyl-2,6-dimethoxy-
33	14.064	45631	0.45	1,3-Cyclohexanediol, 2,5-dimethyl-2-nitro-, monoacetate (
34	14.155	96837	0.96	3-Hydroxy-4-methoxybenzoic acid
35	14.596	129665	1.29	1(2H)-NAPHTHALENONE, 3,4,5,6,7,8-HEXAHYDRO-
36	14.650	40467	0.40	3H-3,10A-METHANO-1,2-BENZODIOXOCIN-3-OL, OC
37	15.118	468988	4.65	1,3,4,5-TETRAHYDROXY-CYCLOHEXANECARBOXY
38	15.462	90659	0.90	13-DOCOSENOIC ACID
39	15.890	24137	0.24	INGOL-12-ACETAT
40	15.973	128029	1.27	TETRAHYDROEDULAN C
41	16.035	38984	0.39	1b,4a-Epoxy-2H-cyclopenta[3,4]cyclopropa[8,9]cycloundе
42	16.095	164494	1.63	7-Oxabicyclo[4.1.0]heptan-3-ol, 6-(3-hydroxy-1-butenyl)-1
43	16.225	58686	0.58	6-Hydroxy-4,4,7a-trimethyl-5,6,7,7a-tetrahydrobenzofuran

Peak#	R.Time	Area	Area%	Name
44	16.305	119657	1.19	2-Hexadecene, 3,7,11,15-tetramethyl-, [R-[R*,R*-(E)]]-
45	16.342	130081	1.29	2,6,10-TRIMETHYLUNDECAN-(5E)-2,5,9-TRIEN-4-ON
46	16.548	207791	2.06	1-OXASPIRO[2.5]OCTAN-4-ONE, 2,2-DIMETHYL-
47	16.711	1195261	11.86	PLUCHIDIOL
48	16.994	337440	3.35	Cyclo(L-prolyl-L-valine)
49	17.155	168208	1.67	2-Methoxy-4,4-dimethyl-2-cyclohexen-1-one
50	17.508	103555	1.03	2H-Pyran, tetrahydro-4-methyl-2-(2-methyl-1-propenyl)-
51	17.828	216212	2.14	3,3-Dimethylglutaric acid
52	17.975	116924	1.16	l-Leucine, N-cyclopropylcarbonyl-, dodecyl ester
53	18.011	93358	0.93	7-Oxabicyclo[4.1.0]heptan-3-ol, 6-(3-hydroxy-1-butenyl)-1
54	18.100	28610	0.28	Diphenyl sulfone
55	18.148	159968	1.59	3-ISOBUTYLHEXAHYDROPYRROLO[1,2-A]PYRAZIN
56	18.285	52421	0.52	2-((3-Methylbutan-2-yloxy)carbonyl)benzoic acid
57	18.347	411409	4.08	n-Hexadecanoic acid
58	20.051	555959	5.51	XANTHAUMIN
59	20.857	63162	0.63	3-METHYLENEBICYCLO[3.2.1]OCT-6-EN-8-OL
60	21.782	97329	0.97	(.+/-.)-Marmesin
61	22.283	71655	0.71	Pyrrolo[1,2-a]pyrazine-1,4-dione, hexahydro-3-(phenylmet
62	23.551	46801	0.46	Silane, dimethyl(2-methylphenoxy)heptyloxy-
63	29.063	110091	1.09	CHOLESTA-4,6-DIEN-3-OL, (3.BETA.)-
64	29.345	137438	1.36	Lup-20(29)-en-3-ol, acetate, (3.beta.)-
		10081702	100.00	

Dried powder of New-fangled stem buds of *Ficus religiosa*

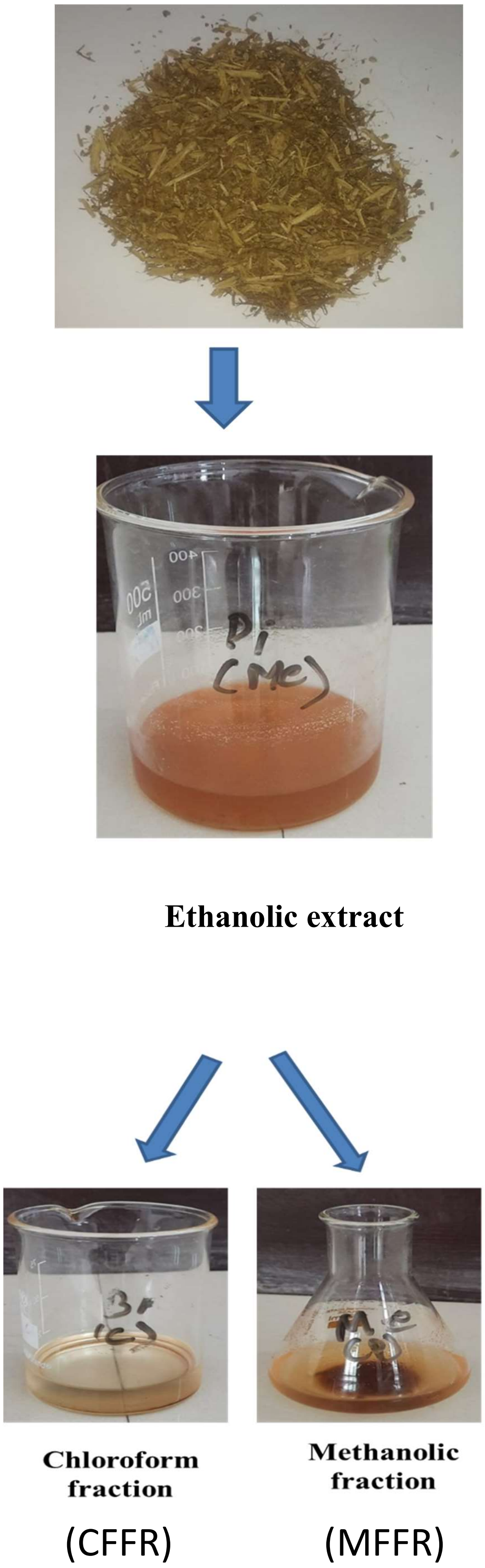

Thrombolytic activity

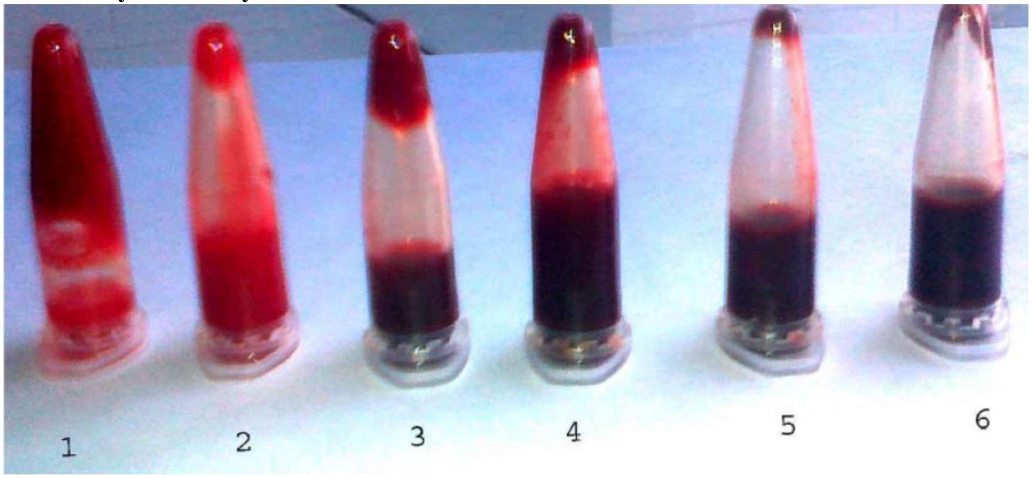

Thrombolytic activity can be clearly observed. Tube no. 1 is negative control to which Normal Saline (No cloth was observed) In tube number 2 is positive control to which 0.5 ml 5K IU SK (Clear clot lysis can be seen) Other tube contains different concentration of MFFR

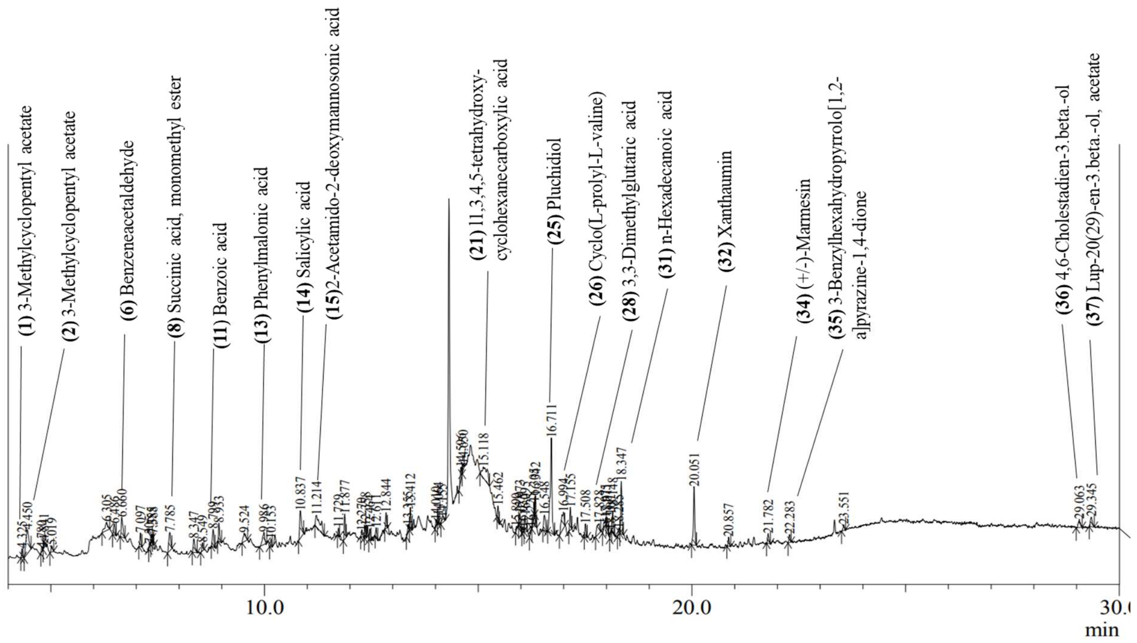

Fig. GC-MS chromatogram of methanol fraction of new-fangled stem buds of *Ficus religiosa*
